# A Gene Expression Screen in *Drosophila melanogaster* Identifies Novel JAK/STAT and EGFR Targets During Oogenesis

**DOI:** 10.1534/g3.118.200786

**Published:** 2018-11-01

**Authors:** Julia Wittes, Trudi Schüpbach

**Affiliations:** Department of Molecular Biology, Princeton University, Princeton, NJ 08544

**Keywords:** EGFR, JAK/STAT, oogenesis, AdamTS-A, *Drosophila*, Sema1b

## Abstract

The Janus Kinase/Signal Transducer and Activator of Transcription (JAK/STAT) and epidermal growth factor receptor (EGFR) signaling pathways are conserved regulators of tissue patterning, morphogenesis, and other cell biological processes. During *Drosophila* oogenesis, these pathways determine the fates of epithelial follicle cells (FCs). JAK/STAT and EGFR together specify a population of cells called the posterior follicle cells (PFCs), which signal to the oocyte to establish the embryonic axes. In this study, whole genome expression analysis was performed to identify genes activated by JAK/STAT and/or EGFR. We observed that 317 genes were transcriptionally upregulated in egg chambers with ectopic JAK/STAT and EGFR activity in the FCs. The list was enriched for genes encoding extracellular matrix (ECM) components and ECM-associated proteins. We tested 69 candidates for a role in axis establishment using RNAi knockdown in the FCs. We report that the signaling protein Semaphorin 1b becomes enriched in the PFCs in response to JAK/STAT and EGFR. We also identified *ADAM metallopeptidase with thrombospondin type 1 motif A* (*AdamTS-A*) as a novel target of JAK/STAT in the FCs that regulates egg chamber shape. *AdamTS-A* mRNA becomes enriched at the anterior and posterior poles of the egg chamber at stages 6 to 7 and is regulated by JAK/STAT. Altering *AdamTS-A* expression in the poles or middle of the egg chamber produces rounder egg chambers. We propose that *AdamTS-A* regulates egg shape by remodeling the basement membrane.

An important biological question is how signaling pathways promote tissue patterning and morphogenetic change. Many pathways carry out these functions by transcriptionally activating or repressing target gene expression. In many developmental and disease contexts, specific signaling pathways have been implicated, but it is not yet understood which transcriptional targets they act on to affect these processes, or how these transcriptional changes are translated into changes in cell and tissue morphology. The goal of this study was to identify novel transcriptional targets of JAK/STAT and EGFR/Ras/MAPK signaling. These conserved signaling pathways regulate normal cell differentiation and proliferation, but can also promote oncogenic transformation, tumor development, and metastasis in disease contexts (Voldborg *et al.* 1997; Hou *et al.* 2002; Arbouzova and Zeidler 2006). Both pathways contribute to the development of certain cancers, such as primary intestinal T-cell lymphomas (Nicolae *et al.* 2016) and hepatocellular carcinomas (Calvisi *et al.* 2006) and it is thought that combined pathway inhibition may therefore be more effective than inhibiting either pathway on its own in some disease contexts (Winter *et al.* 2014). These pathways can also function synergistically to regulate differentiation and morphogenesis during normal cell differentiation and morphogenesis.

The *Drosophila melanogaster* egg chamber is a well-characterized system for studying how signaling pathways specify cell fates and effect morphogenetic change (Horne-Badovinac and Bilder 2005). Egg chambers undergo a highly stereotyped developmental progression that is divided into 14 stages (Spradling 1993). Egg chambers contain two main cell types: somatic epithelial cells, called follicle cells (FCs), which surround the egg chamber in a monolayer; and germline cells, which generate the future egg. Egg chambers originate from a structure called the germarium, which contains the germline stem cells and follicle stem cells. Germline stem cells divide asymmetrically to produce daughter cells called cystoblasts. These divide four times to give rise to a cluster of 16 cells, 15 of which will become nurse cells and one of which will differentiate into the oocyte. Once the 16-cell cluster is formed, follicle cells surround the cluster in a monolayered epithelium to generate the egg chamber (Spradling 1993). Egg chambers are connected to each other by special follicle cells called stalk cells as they grow. A string of egg chambers, surrounded by a muscle sheath, is collectively termed an ovariole and can be thought of as an assembly line that produces mature eggs.

During FC development, signaling generates FC sub-populations with particular functions during oogenesis. If these cell types are not properly specified spatially and temporally, morphogenesis and/or patterning are disrupted in the egg chamber, and later, in the embryo (Berg 2005). The JAK/STAT and EGFR pathways play crucial roles in the patterning of the FCs. Special follicle cells called the polar cells, which can be thought of as signaling hubs and are located at the anterior and posterior ends of each egg chamber, secrete the JAK/STAT signaling ligand Unpaired (Upd; McGregor *et al.* 2002; Figure 1A). During stages 3 to 6, JAK/STAT signaling becomes activated in a gradient in the FCs at the egg chamber poles. In the anterior, JAK/STAT activity specifies the anterior follicle cell (AFC) fate. The future posterior follicle cells (PFCs) are exposed to Upd from the polar cells as well as Gurken, the active ligand for the EGFR, from the oocyte (Figure 1A,B). Together, JAK/STAT and EGFR activity produce the posterior follicle cell (PFC) fate (Xi *et al.* 2003). Previous work has demonstrated that by removing JAK/STAT or EGFR it is possible to disrupt PFC formation, and that ectopic activity of both pathways in the follicle cells can induce ectopic PFCs (Xi *et al.* 2003; Fregoso Lomas *et al.* 2016). In the *Drosophila* egg chamber, EGFR signaling activates the conserved MAPK/ERK signaling cascade (Schnorr and Berg 1996); consequently, disrupting the components of this cascade can also interfere with PFC specification.

**Figure 1:**
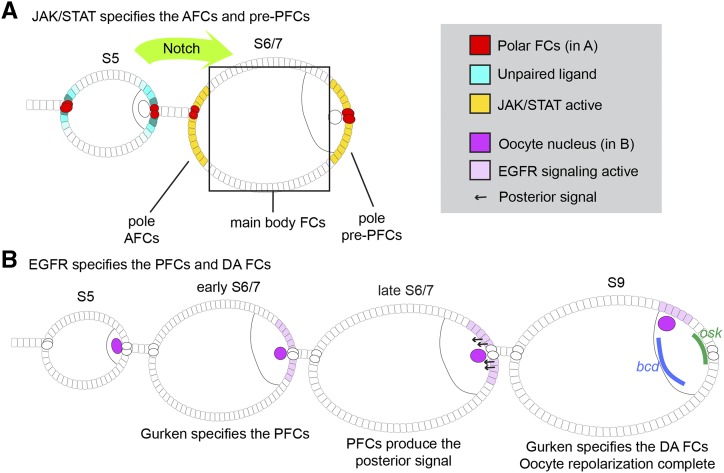
JAK/STAT and EGFR signaling pattern the follicle cells and establish the A/P axis of the developing *Drosophila* egg. A) The polar cells (red) secrete the JAK/STAT signaling ligand Unpaired (cyan). Unpaired activates JAK/STAT signaling activity in the follicle cell (FC) poles (yellow) by stage 5 (S5) to specify the AFCs and presumptive PFCs (pre-PFCs). Main body FCs are indicated. FCs become competent to respond to the JAK/STAT signal at stage 6, following Notch signaling (light green). B) Gurken/EGFR signaling (light purple) specifies the PFCs at stage 6/7 (S6/7). Once specified, the PFCs signal (arrows) to direct oocyte repolarization. During repolarization, the oocyte nucleus (magenta) migrates to the dorsoanterior (DA) corner, *bicoid* RNA (*bcd*, blue) accumulates at the anterior, and *oskar* RNA (*osk*, green) localizes to the posterior. At stage 9 (S9), Gurken/EGFR signaling specifies the DA follicle cells.

The PFCs play a key role in the establishment of the embryonic body axes. Prior to stage 6/7, the oocyte microtubules (MTs) are organized with the minus ends at the posterior cortex (Theurkauf *et al.* 1992). At stage 6/7, shortly after the PFCs are specified, they signal to the oocyte to trigger the reorganization of the oocyte cytoskeleton (González-Reyes *et al.* 1995; Roth *et al.* 1995; Figure 1B). This event is known as oocyte repolarization, and the signaling event that precedes it is referred to as posterior signaling. If posterior signaling occurs normally, the oocyte microtubules become polarized by stage 9 such that the minus ends are oriented toward the anterior of the egg chamber (Clark *et al.* 1994; Cha *et al.* 2002). During this process, microtubules push the oocyte nucleus anteriorly (Zhao *et al.* 2012). Oocyte repolarization has important implications for the development of the embryo because it directs the localization of the anterior determinant, *bicoid* (*bcd*), and the posterior determinant, *oskar* (*osk*) (González-Reyes *et al.* 1995; Roth *et al.* 1995; Figure 1B). These RNAs encode proteins that pattern the head and tail structures in the embryo, and accordingly, if they are not localized properly, the head or tail structures do not form correctly (Frohnhöfer and Nüsslein-Volhard 1986; Lehmann and Nüsslein-Volhard 1986; Berleth *et al.* 1988; Kim-Ha *et al.* 1991).

In addition to specifying posterior follicle cell fates, JAK/STAT and EGFR regulate additional cell and tissue morphologies in the egg chamber. EGFR signaling specifies the dorsoanterior (DA) follicle cells (Figure 1B), which migrate and change shape during late oogenesis to form the tubular dorsal appendages (Price *et al.* 1989; James *et al.* 2002; Osterfield *et al.* 2013). JAK/STAT signaling is required for the specification and proper morphology of the stalk cells, for specification and migration of the border cells, and was recently found to regulate the overall shape of the egg chamber (Silver and Montell 2001; McGregor *et al.* 2002; Baksa *et al.* 2002; Crest *et al.* 2017).

To understand how the JAK/STAT and EGFR pathways pattern the egg chamber and in particular, how they promote posterior signaling, we used gene expression profiling to identify genes transcribed downstream of these pathways. We used genetic tools to ectopically express the JAK/STAT signaling ligand Upd (*UAS-upd*; Tulina and Matunis 2001) and a constitutively active (CA) version of EGFR (*UAS-λtop*; Queenan *et al.* 1997) in the FCs to generate ectopic PFCs. Using microarrays, we identified 317 genes whose expression was enriched under these conditions. Candidate genes that encode cell-surface expressed or secreted (CSS proteins), transcription factors, signaling proteins, ECM proteins and some proteins of unknown function were selected for further investigation. RNAi knockdown was used to test 69 of these candidates for a role in oocyte repolarization. By this approach, we identified a new PFC-enriched protein, Sema1b, which requires both JAK/STAT and EGFR activity for its accumulation in the PFCs. The microarray dataset was also enriched for extracellular matrix (ECM) proteins; therefore, we used RNAi knockdown to study the consequences of disrupting several ECM proteins in the FCs. By this approach, we discovered a novel role for the metalloprotease AdamTS-A during oogenesis. Our results show that *AdamTS-A* is expressed in the AFCs and PFCs downstream of JAK/STAT signaling and is required for egg chamber elongation in a manner that appears to require its protease activity. To our knowledge, our work is the first to describe a metalloprotease that regulates *Drosophila* egg chamber shape. We propose that AdamTS-A helps to remodel the basement membrane at the egg chamber poles to promote elongation.

## Materials and Methods

### Fly strains and genetics

The following stocks were obtained from the Bloomington Drosophila Stock Center (BDSC): *tub:Gal80^TS^* (BL-7019), *sema-1a^P1^* (BL-11097), and *AdamTS-A^MI14156^* (BL-61726). *AdamTS-A* RNAi lines (VDRC-33347 and VDRC-110157) were ordered from the Vienna Drosophila Stock Center (VDRC). *Sema1b-Venus^CPTI003971^* (DGGR-115463) and *Sema1b-GAL4^NP1166^* (DGGR-103911) are from the Kyoto Stock Center. *UAS-upd* was a gift from Doug Harrison. *UAS-λtop4.4* is described in Queenan *et al.* 1997. The *hop* RNAi line used in this study (BL-32966; Figures 3F, 4C, 6D and 6J-L) is described in Recasens-Alvarez *et al.* (2017). The *UAS-upd*, *UAS-λtop* recombinant chromosome was generated by meiotic recombination. *mirr-GAL4*; *tub:GAL80^TS^* and *fru-GAL4*; *tub:GAL80^TS^* (Crest *et al.* 2017), as well as *tj-GAL4* were gifts from David Bilder. *GR1-GAL4* is described in Gupta and Schüpbach, 2003. *UASp-eGFP-48C* was a gift from Elizabeth Gavis. *AdamTS-A^KO^* was a gift from Afshan Ismat (Ismat *et al.* 2013). *AdamTS-A^rnwy1^* was a gift from James Skeath (Skeath *et al.* 2017). *staufen-GFP* (Martin *et al.* 2003) was a gift from Daniel St Johnston; the original insertion was mobilized to generate an insertion on the X-chromosome using *Δ2-3* transposase. *Oregon R* (*OreR*) flies were used as wild type (WT). RNAi lines used in the screen were from the BDSC or VDRC and their stock numbers are provided in Supplemental Material, File S5.

Full experimental genotypes and incubation temperatures are reported in File S7.

### Microarray preparation and data analysis

Ovaries from females fed yeast for two days were dissected in PBS and flash frozen in liquid nitrogen. Ovaries from WT (*OreR*) females were dissected in three batches, frozen and pooled to generate a reference pool of RNA. 5-10 ovary pairs from 5-8 day old *GAL80^TS^*; *GR1-GAL4*/*UAS-upd*, *UAS-λtop* and *GAL80^TS^*; *GR1-GAL4*/*TM6* egg chambers were pooled for each preparation; this minimizes biological variation and ensured a sufficient quantity of RNA was collected for microarray analysis. RNA was extracted using TRIzol reagent (ThermoFisher), cleaned using an RNeasy kit (Qiagen), and DNase-treated with the Turbo DNA-free kit (ThermoFisher) according to the manufacturer’s instructions. RNA concentrations were then measured by a NanoDrop spectrophotometer. To confirm that RNA was free of degradation, RNA used for microarray analysis was analyzed by non-denaturing agarose gel electrophoresis.

Each labeling reaction was performed using 322 ng of RNA. Reference RNA samples were labeled with Cy3-CTP and all other samples were always labeled with Cy5-CTP. Labeling and hybridization were performed using a Quick Amp two-color labeling kit (Agilent), and a labeling protocol optimized by Maitreya Dunham and Bing He (He *et al.* 2012). Labeled cRNA was purified with a RNeasy column (Qiagen) and hybridized at 65° for 17 h, and then washed using Agilent’s proprietary buffers according to standard protocols. The microarray slides used were 4X44K *Drosophila* oligo arrays (print 021791), based on V2 of the *Drosophila* genome. Slides (6 arrays on 3 slides) were scanned immediately following washing to minimize sample degradation. Scans were made at a resolution of 5μ/pixel using an Agilent G2505C scanner and Agilent Feature Extraction version 11.0.11. Raw data were stored at Princeton’s PUMA database (http://puma.princeton.edu/). Bioconductor’s Limma package for R (www.bioconductor.org) was used to Loess normalize, background correct (by the normexp method), and quantile normalize, and finally, to identify differentially expressed probes. False discovery rate (FDR) estimates were calculated in Limma using the Benjamini-Hotchberg method (Benjamini and Hotchberg 1995).

To generate a list of all genes upregulated in *GAL80^TS^*; *GR1-GAL4/UAS-upd*, *UAS-λtop* ovaries as compared to control *GAL80^TS^*; *GR1-GAL4*/*TM6* ovaries, expression thresholds and a p-value threshold were selected. Transcripts included in the final 317 gene dataset were upregulated at least twofold with a false discovery rate threshold of 0.05. If more than one probe was present per gene in the arrays, or if duplicate probes were present in the arrays, values corresponding to the most highly significant data were included in Tables 1-2. Raw and normalized microarray data have been deposited at GEO.

**Table 1:** Microarray Validation. PFC-expressed genes are enriched in the microarray dataset. Genes that are known transcriptional targets of the JAK/STAT or EGFR pathways are indicated. Fold enrichment and adjusted P-values for differential expression are also provided

Gene ID	Fold change	Adj. P- value	JAK/ STAT	EGFR	Reference(s)
*aos*	*1.54	2.60E-03		x	Queenan *et al.* 1997, Zhao and Bownes 1999
*bib*	2.98	7.07E-05			Ruohola *et al.* 1991
*CG11275*	17.2	5.03E-06			Jambor *et al.* 2015
*dome*	2.37	8.44E-05	x		Ghiglione *et al.* 2002, Xi *et al.* 2003
*H15*	7.45	†8.57E-01	x	x	Fregoso Lomas *et al.* 2013, Fregoso Lomas *et al.* 2016
*ImpL2*	5.78	1.56E-05	x	x	Jordan *et al.* 2005
*jim*	2.98	1.48E-03		x	Doerflinger *et al.* 1999
*kek1*	2.01	1.38E-03		x	Musacchio and Perrimon, 1996, Queenan *et al.* 1997
*mid*	2.09	1.75E-03	x	x	Fregoso Lomas *et al.* 2013, Fregoso Lomas *et al.* 2016
*pnt*	2.58	2.93E-04	x	x	Morimoto *et al.* 1996, Xi *et al.* 2003, Flaherty *et al.* 2009
*Socs36E*	15.5	3.38E-05	x		Rawlings *et al.* 2004
*Stat92E*	*1.99	1.34E-03	x		Xi *et al.* 2003, Assa-Kunik *et al.* 2007
*sty*	4.23	3.32E-05		x	Reich *et al.* 1999
*tsl*	3.86	3.91E-04	x		Savant-Bhonsale and Montell 1993, Furriols *et al.* 2007

* Below the twofold expression threshold but above the P-value threshold.

Below the P-value threshold but above the twofold expression threshold.

**Table 2:** Genes encoding ECM and ECM-associated proteins identified in the microarray dataset. Each protein’s role or function is briefly summarized, and fold changes and adjusted P-values for differential expression are provided

Gene	Fold change	Adj. P value	Role
*upd*	63.1	3.99E-08	Ligand
*Mmp2*	5.54	1.36E-04	Protease
*SPARC*	3.34	7.30E-05	Collagen-binding
*AdamTS-A*	2.84	8.82E-05	Protease
*Ppn*	2.76	8.82E-05	Protease
*scb*	2.49	1.65E-04	Integrin
*trol*	2.49	3.34E-05	HSPG
*m*	2.45	2.40E-04	Secreted or ECM
*LanB1*	2.27	1.92E-03	Laminin
*Fas1*	2.26	5.63E-03	Adhesion
*Mp*	2.23	6.55E-04	Collagen XVIII
*Col4a1*	2.18	8.67E-03	Collagen IV
*LanB2*	2.02	2.58E-03	Laminin

### Immunofluorescence

Females were fed yeast for two days, dissected in room temperature PBS, fixed in 4% PFA for 20 min at room temperature, and washed in PBST (1X PBS with 0.1% Triton X-100; Sigma-Aldrich). Primary antibodies used were rabbit anti-GFP (AB3080P; 1:500; Millipore), guinea pig anti-Midline (1:200, a gift from Laura Nilson; Fregoso Lomas *et al.* 2013) and goat anti-Vasa (dN-13; 1:1000; Santa Cruz Biotechnology). Secondary antibodies used were Alexa Fluor 488, 555, 568 or 647-conjugated (1:400; Life Technologies). DNA was stained with Hoechst (10 μg/mL; Life Technologies). Alexa Fluor 546 Phalloidin (1:500; Life Technologies) or Alexa Fluor 647 Phalloidin (1:250; Life Technologies) were used to stain F-actin. For all experiments, egg chambers were mounted in Aqua-Poly/Mount (Polysciences). All micrographs in this study were taken using a Nikon A1 laser scanning confocal microscope. Images were acquired using a 40X, 1.30 NA Nikon Apo Plan Fluor or a 60X, 1.40 NA Nikon Plan objective. Images were prepared using Fiji and Adobe Photoshop CS6.

### Fluorescent in situ hybridization (FISH)

Ovaries were prepared for FISH as described in Abbaszadeh and Gavis (2016) with two deviations: ovaries were incubated in MeOH at -20° for 15 min and mounted in Aqua-Poly/Mount. FISH probes conjugated to Quasar 570 were ordered from LGC Biosearch Technologies. 5 nmol of lyophilized probes were resuspended in 200 μL of TE Buffer (pH 8.0), and 1 μL of resuspended probe was used for each 100 μL hybridization reaction. A full list of probes used for FISH can be found in File S6.

### RNAi knockdown

To test candidate genes for a role in oocyte repolarization, *stau-GFP*; *tj-GAL4*; *UAS-Dcr2/TM6* virgins were crossed to *UAS-RNAi* males. Crosses were carried out at 25°, and once F1 females hatched, they were incubated at 29° with yeast for two days prior to dissection. Egg chambers were stained with Alexa Fluor 546 Phalloidin and Hoechst. The penetrance of Stau-GFP mislocalization was scored using an EVOS FL epifluorescence microscope (ThermoFisher) at stages 9 and 10 of oogenesis. Egg chambers that appeared to be dying or that could not be accurately staged were not scored. In control egg chambers, Stau-GFP localizes strongly to the posterior of the oocyte at stage 10 of oogenesis. If Stau accumulated at the center of the oocyte, appeared dispersed or in blobs, or only accumulated weakly at the posterior, we considered Stau “mislocalized.” If other oogenesis phenotypes were observed, these were recorded as well. RNAi lines were considered ‘hits’ if they produced ≥50% Stau-GFP mislocalization at stage 10.

A full list of RNAi knockdown reagents is included in File S5, which also reports the penetrance of Stau-GFP mislocalization for each line tested. When possible, we tested multiple RNAi lines for each candidate gene.

### Generation of the Sema1b^KO^ allele

We designed a scheme to generate a functional null *Sema1b* allele by replacing a large portion of the *Sema1b* coding region with a dsRed-containing cassette. ∼1 kb homology arms were designed to flank the gRNA cut site, and cloned into the pHD-dsRed-attP vector. 100 ng/μL of each gRNA plasmid and 500 ng/μL of the HDR plasmid were injected into *w*; *FRT42D*; *nos-Cas9* embryos by Rainbow Transgenic Flies. Mutagenized flies were identified by expression of 3XP3::dsRed in the adult eye. Sequencing was performed to confirm integration of the dsRed-containing cassette at the intended genomic location with removal of the targeted region. We also confirmed by sequencing that the vector was not integrated at the *Sema1b* locus and that the flanking regions were not altered.

### Generating Sema1a^P1^ mutant clones in a Sema1b^KO^ mutant background

Negatively-marked mitotic clones were generated in the FCs by the Flp-FRT system (Golic and Lindquist 1989; Xu and Rubin 1993). The following stocks were crossed to produce clones in a *Sema1b^KO^* mutant background: *w*, *hsFlp*; *Sema1a^P1^*, *FRT40A*, *Sema1b^KO^/CyO* and *ubiGFP*, *FRT40A*, *Sema1b^KO^*.

### Egg chamber aspect ratio calculation

Egg chambers stained with Hoechst and Alexa Fluor 546 Phalloidin were mounted onto glass slides with a coverslip and photographed using an EVOS FL epifluorescence microscope. ImageJ was used to measure egg chambers along the A/P and D/V axes. The aspect ratio is the ratio of the length along the A/P axis to the width, measured along the D/V axis. Egg chambers were only included in this analysis if there were no gaps in the follicular epithelium and if the egg chamber did not appear to be degenerating. Welch’s two-sample *t*-tests were used to compare the aspect ratios, lengths and widths of two groups.

### Data availability

All data necessary to confirm the conclusions presented in this article are represented fully within it and the associated materials. Strains are available upon request. Raw and normalized microarray data were deposited at the Gene Expression Omnibus (GEO) under accession number GSE118881. File S1 contains a list of all microarray probes that pass the fold change and P-value thresholds. The genes in this list are sorted into categories and their function, if known, is summarized. Raw P-values and adjusted P-values are also reported in File S1. Files S2, S3 and S4 contain the result of GO statistical overrepresentation analysis of the microarray dataset using Panther, LAGO, and DAVID, respectively. File S5 contains a list of the genes selected for the RNAi screen as well as Stau-GFP scoring (at stage 10) for each RNAi line tested. File S6 lists the probe sequences used for *AdamTS-A* FISH. File S7 contains a full list of experimental genotypes and incubation temperatures. Table S1 contains quantification associated with Figure 3. Supplemental material available at Figshare: https://doi.org/10.25387/g3.7209809.

## Results

This study aimed to identify targets of the JAK/STAT and EGFR pathways during *Drosophila* oogenesis. The research that established the instructive role of JAK/STAT and EGFR in FC patterning showed that modulating these pathways can interconvert certain sub-populations of FCs. For instance, ectopically expressing EGFR in the anterior is sufficient to convert AFCs into PFC-like cells that morphologically resemble PFCs and express PFC-specific markers (Lee and Montell 1997; Keller Larkin *et al.* 1999; Xi *et al.* 2003). Similarly, if EGFR is lost from the PFCs, they assume an AFC fate and express AFC markers (Roth *et al.* 1995; González-Reyes *et al.* 1995, Xi *et al.* 2003). If Upd is ectopically expressed in the main body follicle cells, they assume an AFC fate (Xi *et al.* 2003).

**Figure 2:**
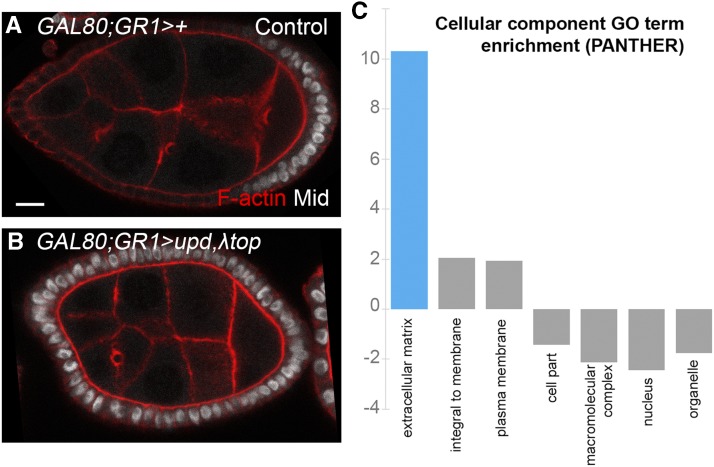
Ectopic expression of JAK/STAT and EGFR in the follicular epithelium of the *Drosophila* egg chamber. A) Midline (Mid, white) protein is expressed specifically in the PFCs in a control (*GAL4* and *GAL80* only) stage 7 egg chamber. F-actin is shown in red. Scale bar is 10 μm. B) Ectopic expression of *upd* (JAK/STAT ligand) and *λtop* (constitutively active EGFR) results in Mid expression in all FCs. A&B both show stage 7/8 egg chambers. The egg chamber in B is not as elongated as the one depicted in A because ectopic expression of *upd* in the FCs disrupts egg chamber elongation (Alégot *et al.* 2018). C) Cellular component gene ontology (GO) terms enriched in egg chambers with ectopic EGFR and JAK/STAT activity. Analysis of the microarray dataset was performed with PANTHER (http://www.pantherdb.org/). Extracellular matrix (ECM) proteins (blue) are overrepresented.

However, few downstream effectors for these pathways have been identified. To identify genes transcriptionally activated by JAK/STAT and EGFR in the FCs, we used the GAL4-UAS system (Brand and Perrimon 1993) to ectopically express JAK/STAT and EGFR pathway components throughout the follicular epithelium. Using the *GR1-GAL4* driver, we ubiquitously expressed the JAK/STAT ligand Unpaired (*UAS-upd*) in the FCs to activate JAK/STAT (Figure S1 shows the *GR1-GAL4* driver’s expression pattern). To activate EGFR signaling in the FCs, we expressed a dominant active EGFR using *UAS-λtop* (Queenan *et al.* 1997). Previous work has shown that ectopic expression of these two signaling components is sufficient to convert AFCs and main body follicle cells into PFCs (Fregoso Lomas *et al.* 2016). However, to confirm that this was the case, we stained control and ectopic expression egg chambers using an antibody against Midline (Mid), which is expressed specifically in the PFCs (Fregoso Lomas *et al.* 2013; Figure 2A). Ectopic JAK/STAT and EGFR activity successfully converted all FCs into PFC-like cells with detectable Mid protein expression (Figure 2B). Microarrays were then used to identify transcripts expressed in response to ectopic EGFR and/or JAK/STAT signaling.

### Ectopic expression of EGFR and JAK/STAT signaling components identifies both known and novel transcriptional targets

We found 589 microarray probes representing 317 unique genes to be upregulated at least twofold in ectopic expression ovaries using an adjusted P-value threshold of 0.05 (File S1). Of the 14 genes that are known to date to be transcriptionally enriched in the PFCs, 12 are found in the 317 gene dataset with significant upregulation (Table 1). The other two genes, *argos* (*aos*) and *Stat92E*, were upregulated and passed the FDR threshold, but fell below the twofold expression threshold. We also identified a number of other transcripts that are known JAK/STAT and EGFR signaling targets in other contexts. Several transcripts identified by microarray analysis are known to be required in the PFCs for oocyte repolarization but are not known JAK/STAT or EGFR targets. These include *alpha*- and *beta-spectrin* (Lee *et al.* 1997; Wong *et al.* 2015) and *mbs* (Sun *et al.* 2011). The fact that the microarray dataset includes essentially all of the known PFC-expressed genes validates our microarray dataset and strongly suggests that it should include other, as yet unidentified PFC-expressed transcripts.

To test if the microarray “hit” list was enriched for transcripts encoding proteins involved in particular biological processes or localized to certain parts of the cell, we used the PANTHER online database (www.pantherdb.org/) for statistical overrepresentation tests (Mi *et al.* 2013). Notably, extracellular matrix (ECM) proteins are 10.31-fold statistically overrepresented in the microarray dataset (FDR = 3.41x10^−3^, Fisher’s exact test; Figure 2C). We also performed complementary gene ontology (GO) analysis using LAGO and DAVID, which also suggested an enrichment of ECM proteins. GO analysis data can be found in the supplement (Files S2-S4). ECM proteins present in the 317 transcript dataset include SPARC, Collagen IV (Col4a1), Terribly reduced optic lobes (Trol, the *Drosophila* homolog of Perlecan), the collagen Multiplexin (Mp); the laminins Laminin B1 (LanB1) and Laminin B2 (LanB2); Fasciclin 1 (Fas1) and the αPS3 integrin Scab (Scb), Kekkon-1 (Kek1), CG7702, the matrix-modifying protein Sulf1, and the proteases Papilin (Ppn), Matrix metalloprotease 2 (Mmp2), and AdamTS-A (Table 2).

We next tested 69 candidate genes from the microarray for a requirement in posterior signaling during oogenesis. RNAi knockdown was used to disrupt candidate gene expression in the FCs, and a *staufen-GFP* transgene (*stau-GFP*; Martin *et al.* 2003) was used to visualize oocyte polarity at stages 9-10 of oogenesis. We focused on genes encoding cell surface and secreted (CSS; Kurusu *et al.* 2008) proteins, ECM proteins, cytoskeleton interactors and transcription factors because these classes of proteins seemed the most likely to be involved in the differentiation of the PFCs or their function in signaling to repolarize the oocyte. We generated the stock *stau-GFP*; *tj-GAL4*; *UAS-Dcr2* and crossed virgins to males carrying UAS-RNAi constructs or RNAi controls. *tj-GAL4* and *UAS-Dcr2* facilitate candidate gene knockdown in the FCs, and the *stau-GFP* transgene is a useful readout for oocyte repolarization (Martin *et al.* 2003; Dietzl *et al.* 2007). *traffic jam-GAL4* (*tj-GAL4*) has been used successfully in previous RNAi screens in the FCs (Handler *et al.* 2013; Berns *et al.* 2014) and it produces expression in the FCs throughout their development (Olivieri *et al.* 2010). An RNAi line was considered a “hit” if it produced a Stau-GFP mislocalization phenotype of ≥50% penetrance at stage 10. 11 RNAi lines targeting nine different genes were counted as “hits” (Table 3). We tested ten RNAi lines targeting seven known posterior signaling genes as positive controls (File S5). Of these ten lines, three disrupted oogenesis before stage 10 (lines targeting *ERK*, *EGFR* and α*-Spec*), and two produced a Stau-GFP mislocalization phenotype (an α*-Spec* RNAi line and a *MEK* RNAi line; see Table 3). The remainder of the positive control lines tested fell below the 50% mislocalization cutoff (for stage 10) and were not scored as ‘hits’. As an additional positive control, we performed a cross with a dominant negative EGFR transgene (*UAS-DN-DER*; Buff *et al.* 1998). This positive control successfully resulted in Stau mislocalization. Based on the results of our control experiments, we expected false negatives in the RNAi knockdown screen and that some posterior signaling genes might cause a premature termination of normal oogenesis before stage 10.

**Table 3:** RNAi knockdown and accompanying control lines characterized as ‘hits.’ Lines where >50% of stage 10 egg chambers showed Stau-GFP mislocalization were considered ‘hits’

Gene	Stock	% Stau misloc S10	Cellular function
**Negative control lines**			
*N/A*	*OreR*	3	N/A
*N/A*	V60101	0	KK library control
**Positive control lines**			
*EGFR*	*UAS-DN-DER*	100	EGFR pathway
*α-spec*	BL42801	74	Spectrin
*MEK*	BL32920	68	EGFR pathway
**RNAi screen ‘hits’**			
*CG6340*	V34160	100	Unknown
*Csk*	V109813	98	Kinase activity
*Csk*	V32877	96	Kinase activity
*CG13510*	V28434	95	Unknown
*CG8547*	V110523	92	Unknown
*ImpL3*	V110190	92	Lactate dehydrogenase
*Sema1b*	V107233	88	Signaling ligand
*pico*	V16371	86	Actin regulator
*Sema1b*	BL28588	71	Signaling ligand
*CG13506*	V14127	70	Unknown
*Ppn*	V16523	55	ECM protease

Only one gene encoding a CSS or signaling protein was identified as a ‘hit’ in the RNAi knockdown screen: the putative signaling ligand Semaphorin 1b (Sema1b; Table 3). Posterior signaling defects were also seen for several RNAi knockdown lines targeting genes of unknown function, including *Ecdysone-induced protein L3* (*ImpL3*), which encodes the metabolic enzyme lactate dehydrogenase, *pico*, an actin-regulator, and *Papilin* (*Ppn*), a matrix metalloprotease. *C-terminal Src kinase* (*Csk*), encodes a tyrosine kinase that is best studied for its role in inhibiting the activity of the two *Drosophila* Src kinases, Src42A and Src64B (Pedraza *et al.* 2004). *Csk* has been shown to genetically interact with components of JAK/STAT, Hippo and EGFR/Ras signaling pathways in *Drosophila* (Stewart *et al.* 2003; Read *et al.* 2004; Hirabayashi *et al.* 2013; Kwon *et al.* 2015). We also fortuitously found that two RNAi lines targeting *AdamTS-A* resulted in the production of abnormally round egg chambers. AdamTS-A is a member of the AdamTS family of metalloproteases, and it has been reported to be expressed in a number of migratory tissues in the *Drosophila* embryo, including the caudal visceral mesoderm, trachea, and hemocytes. Proteolytic targets of AdamTS-A have not yet been definitively reported, but it is thought to modify ECM components in the larval CNS and to regulate cell-matrix adhesion in the developing salivary gland (Ismat *et al.* 2013; Skeath *et al.* 2017). We selected Sema1b and AdamTS-A for further study based on their RNAi knockdown phenotypes, expression patterns (see below), and putative functions.

### sema1b mRNA and Sema1b protein are enriched in the polar cells and PFCs

**Figure 3:**
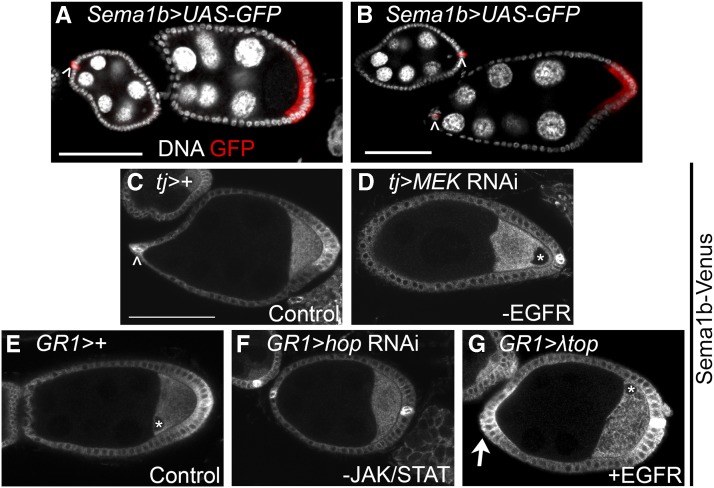
*Sema1b* RNA and protein are enriched in the PFCs and polar FCs. Sema1b protein accumulates in FCs with both JAK/STAT and EGFR signaling. A,B) To visualize *Sema1b* expression during oogenesis, *Sema1b-GAL4* was used to drive a *UASp-eGFP* reporter. *Sema1b* was observed in the polar follicle cells (arrowheads) and in the PFCs beginning at stage 7 (red). Polar cells outside of this *z*-plane also express the reporter. C,E) *Sema1b-Venus* protein trap was used as a reporter for Sema1b protein localization during oogenesis. Sema1b-Venus was detected in the polar follicle cells (arrowhead) and PFCs. Stage 8 is pictured in panels C-E. C) Control (*GAL4* only) for comparison to D. D) When *MEK* RNAi knockdown was very effective in disrupting PFC differentiation, as evidenced by the loss of oocyte nuclear migration (*), Sema1b-Venus enrichment in the PFCs was also disrupted. E) Control (*GAL4* only) for comparison to F and G. F) Disruption of JAK/STAT signaling using *hop* knockdown disrupts Sema1b-Venus accumulation in the PFCs. G) Ectopic expression of constitutively active EGFR (*λtop*) produces ectopic Sema1b-Venus accumulation in the AFCs (arrow) and occasionally causes a loss of Sema1b enrichment in the PFCs (data not shown). Asterisks (*) mark visible oocyte nuclei. Scale bars represent 50 μm. Table S1 includes quantification of Sema1b-Venus accumulation in the AFC and PFCs and oocyte nuclear migration defects to accompany panels C-G. An anti-GFP antibody was used to detect UAS-GFP in panels A&B and Sema1b-Venus in panels C-G.

Microarray analysis demonstrated a 3.6-fold increase in *Sema1b* transcript in ovaries with ectopic PFCs. To visualize the expression pattern of *Sema1b* mRNA in the ovary, we used the GAL4 driver *Sema1b^NP1166^* (Figure S2) to drive the expression of a GFP reporter (*UASp-EGFP*). GFP expression was largely confined to the polar follicle cells and PFCs (Figure 3A,B). To test whether Sema1b protein localizes to the PFCs, as the previous result suggests, we used the protein trap line *Sema1b^CPTI003971^*, where Venus is spliced into the Sema1b protein between exons 2 and 3 (Figure S2). We observed enrichment of Venus in the polar FCs and PFCs at mid-oogenesis (Figure 3C). Together these data suggest that *Sema1b* is enriched in the PFCs and polar cells at both the mRNA and protein levels.

### Sema1b protein enrichment in the PFCs requires JAK/STAT and EGFR signaling

Semaphorins are signaling proteins that can signal by physically interacting with other Semaphorins or with a closely-related family of signaling proteins called Plexins. Semaphorins and Plexins share an approximately 500 amino acid domain called a Sema domain, which they use to interact (Kolodkin *et al.* 1993). Semaphorins come in transmembrane, secreted, and membrane-linked (GPI-anchored) forms, and all Plexins are transmembrane proteins (Yu and Kolodkin 1999). There are five known Semaphorins and two Plexins encoded in the fly genome. To test whether Sema1b expression is regulated by JAK/STAT or EGFR signaling, we examined the levels of Sema1b-Venus in different knockdown conditions. *MEK* RNAi knockdown, which was used to reduce EGFR signaling, disrupted Sema1b-Venus enrichment in the PFCs. A strong disruption of EGFR signaling interferes with the migration of the oocyte nucleus to the dorsoanterior corner (Neuman-Silberberg and Schüpbach 1996; Figure 3D). The GAL4 system used to express the *MEK* RNAi construct produced a variable effect; some egg chambers displayed a strong phenotype and produced a failure of oocyte nuclear movement, and others did not. In egg chambers where the oocyte nucleus failed to move, Sema1b-Venus levels were substantially reduced in the PFCs (Figure 3D). RNAi knockdown of the kinase *JAK/hopscotch (hop)* also strongly disrupted Sema1b-Venus in the PFCs (compare Figure 3E&F). Finally, we observed that if EGFR was ectopically activated in the FCs by expressing *UAS-λtop*, Sema1b-Venus was observed in the AFCs, where endogenous JAK/STAT signaling is present, but not in the main body follicle cells (Figure 3G). Taken together, these data indicate that both JAK/STAT and EGFR signaling promote Sema1b expression in the PFCs. The penetrance of Sema1b localization and nuclear migration defects for each genetic condition in Figure 3C-G is reported in Table S1.

### Sema1b RNAi knockdown produces oocyte repolarization defects (Figure 4A,B)

**Figure 4:**
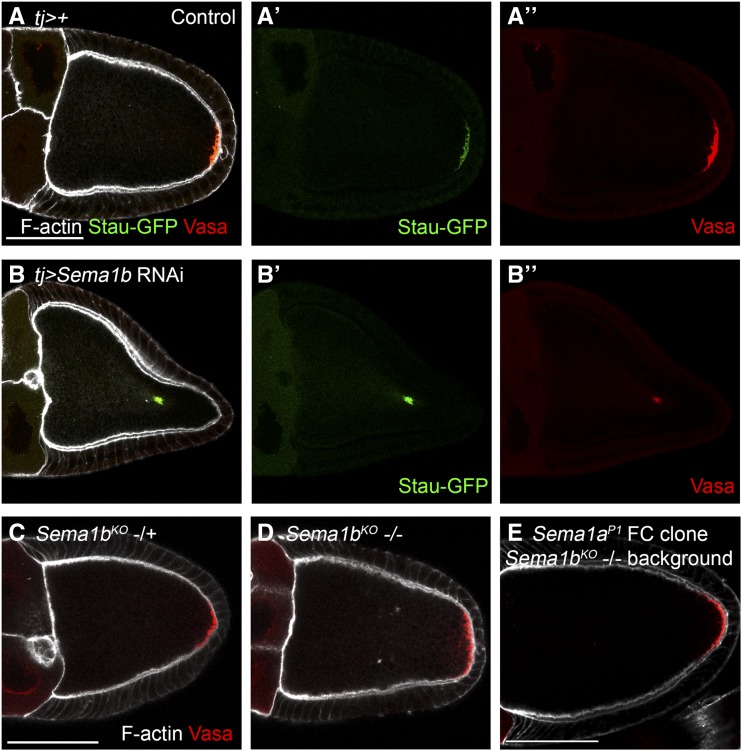
*Sema1b* knockdown disrupts oocyte repolarization; however, *Sema1b* mutants do not appear to have oocyte repolarization defects. A-A’’) Stau-GFP (green) and Vasa (red) both localize to the oocyte posterior in Control (*GAL4* only) stage 10 egg chambers. F-actin is in white. B-B’’) *Sema1b* RNAi produces mislocalization of both Stau-GFP and Vasa to the center of the oocyte at stage 10. C&D) Egg chambers heterozygous or homozygous for *Sema1b^KO^* (a deletion allele) do not show oocyte repolarization defects by Vasa (red) immunostaining. F-actin is in white. E) *Sema1a^P1^* follicle cell clones were generated in a *Sema1b^KO^* mutant background. All FCs in this egg chamber are doubly mutant (a total FC clone is pictured). Vasa localization in these egg chambers is normal. All scale bars represent 50 μm.

Using Stau-GFP as an oocyte polarity marker, we observed posterior signaling defects for two overlapping *Sema1b* RNAi knockdown lines (VDRC-107233/line 1 and BL-32877/line 2). *Sema1b* RNAi line 1 produced Stau-GFP mislocalization with 89% penetrance (n = 116) and line 2 produced Stau-GFP mislocalization with 71% penetrance (n = 24) at stage 10. Control egg chambers, where *Stau-GFP*; *tj-GAL4*; *UAS-Dcr2* were crossed to WT resulted in 3% Stau-GFP mislocalization (n = 98), and the RNAi (KK) library control (VDRC-60101) produced 3% Stau-GFP mislocalization (n = 75). Since *Sema1b* RNAi line 1 produced a stronger phenotype, we further characterized the *Sema1b* RNAi knockdown phenotype using this line. To confirm the Stau-GFP mislocalization phenotype, we performed immunostaining for a second marker of oocyte polarity: the protein Vasa, which localizes to the oocyte posterior at stage 10 in response to posterior signaling (Figure 4A; Hay *et al.* 1988). *Sema1b* RNAi knockdown causes Vasa mislocalization (Figure 4B). As a final readout of oocyte polarity in *Sema1b* RNAi knockdown egg chambers, we used a kinesin-lacZ transgenic reporter. In control stage 9 egg chambers, MTs were correctly polarized and Kinesin-βgal always localized to the posterior of the egg chamber (100%, n = 19; Figure S3A). By contrast, when *Sema1b* was knocked down in the FCs, Kinesin-βgal was frequently mislocalized (78% mislocalization; n = 32; Figure S3B).

### Egg chambers mutant for Sema1b do not display oocyte repolarization defects

To further test whether *Sema1b* is required for oocyte repolarization, we generated a *Sema1b* mutant allele using CRISPR and homology driven repair (HDR). Our approach replaced a large portion of the Sema1b coding region, including the conserved Semaphorin domain, with a dsRed cassette (Figure S2; Gratz *et al.* 2014). The allele was verified by sequencing. Because the signaling domain was removed in the mutant and a frameshift was generated that produced a premature stop codon, the allele is likely a functional, if not protein, null. Given the strong oocyte repolarization defects we observed by RNAi knockdown, we were surprised to find that the allele generated by CRISPR, *Sema1b^KO^*, was homozygous viable and females were fertile. Ovaries from homozygous mutant females appeared normal and Vasa protein localized properly to the posterior (compare Figure 4C&D). Two independently derived *Sema1b^KO^* lines were examined, and neither displayed a Vasa mislocalization phenotype. We also did not observe Vasa mislocalization defects in egg chambers with FC clones mutant for *Sema-1b^KO^* (data not shown). To determine if functional redundancy between the two known type I Semaphorins might account for the lack of defects observed in the *Sema1b^KO^* mutant allele, we generated mutant clones for a lethal *Sema1a* null allele (*Sema1a^P1^*) in a *Sema1b^KO^* homozygous mutant background. We again did not detect any posterior signaling defects; Vasa localized correctly when *Sema1a* and *Sema1b* were both disrupted in the follicle cells (Figure 4E). As discussed later, it is possible that other related proteins, such as other Sema-domain containing proteins, could compensate for the loss of *Sema1b* in the *Sema1b^KO^* mutant.

### AdamTS-A is transcriptionally activated by JAK/STAT in the follicle cells

**Figure 5:**
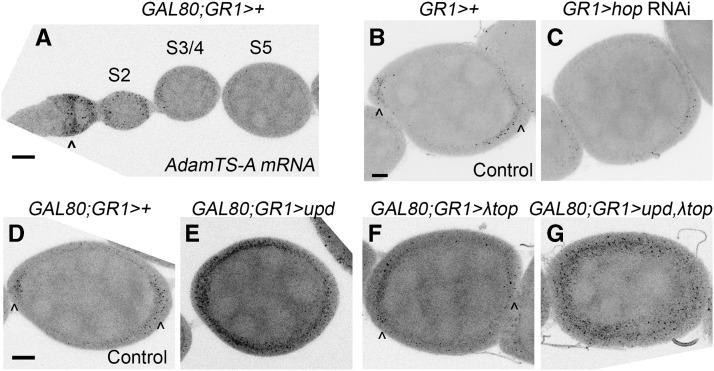
*AdamTS-A* expression is regulated by JAK/STAT signaling. A) Fluorescent *in situ* hybridization (FISH) of control (*GAL4* and *GAL80* only) egg chambers shows *AdamTS-A* expression in the germarium (arrowhead). B-G show stage 6/7 egg chambers. B) Control (*GAL4* only) egg chamber for comparison with C, showing *AdamTS-A* expression in the anterior and posterior follicle cells (AFCs and PFCs) beginning at mid-oogenesis (arrowheads). *AdamTS-A* is present at reduced levels in the main body follicle cells. C) *hop* RNAi in the follicle cells causes a reduction in *AdamTS-A* expression. D) Control (*GAL4* and *GAL80* only) egg chamber for comparison with E-G. Arrowheads indicate AFC and PFC enrichment. E) Ectopic *upd* expression causes increased *AdamTS-A* expression in the FCs. Increased *AdamTS-A* expression in the main body follicle cells is particularly notable. F) Egg chambers expressing constitutively active EGFR (*λtop*) resemble controls. G) Egg chambers co-expressing *upd* and *λtop* show ectopic *AdamTS-A* expression. Panels A-G are maximum intensity projections generated from 21 *z*-slices at 1 μm intervals except for F, which is a projection of 20 z-slices. Scale bars represent 10 μm.

We fortuitously observed that RNAi knockdown of the metalloprotease *AdamTS-A* strongly disrupts egg chamber elongation, a process that is regulated by JAK/STAT signaling (Crest *et al.* 2017; Alégot *et al.* 2018). To determine where *AdamTS-A* is expressed, we used fluorescent *in situ* hybridization (FISH) to examine *AdamTS-A* expression (Figure 5A&D; see File S6 for FISH probes). *AdamTS-A* transcripts are present in FCs early in oogenesis in the germarium. Additionally, by stages 6/7, *AdamTS-A* transcript levels become more enriched in the terminal follicle cells (the AFCs and PFCs) than in the main body follicle cells.

To test whether *AdamTS-A* expression is activated by JAK/STAT or EGFR, we ubiquitously activated both pathways in the FCs and then used FISH to detect any resulting changes in *AdamTS-A* expression. *UAS-upd* expression in the follicle cells caused strong ectopic *AdamTS-A* expression in the main-body follicle cells at stage 6/7 (Figure 5E). *UAS-λtop* expression does not appear to affect *AdamTS-A* in the main body follicle cells (Figure 5F). Coexpression of *UAS-upd* and *UAS-λtop* resembles the *UAS-Upd*-only condition (Figure 5G). If JAK/STAT signaling activates *AdamTS-A* expression in the poles of the egg chamber, then disrupting JAK/STAT should reduce *AdamTS-A* expression. Consistent with this prediction, *hop* RNAi knockdown causes a reduction in *AdamTS-A* expression in the egg chamber poles at stage 6/7 (compare Figure 5B&C). Together, these data suggest that JAK/STAT regulates *AdamTS-A* expression in the FCs.

### AdamTS-A is required for egg chamber elongation

**Figure 6:**
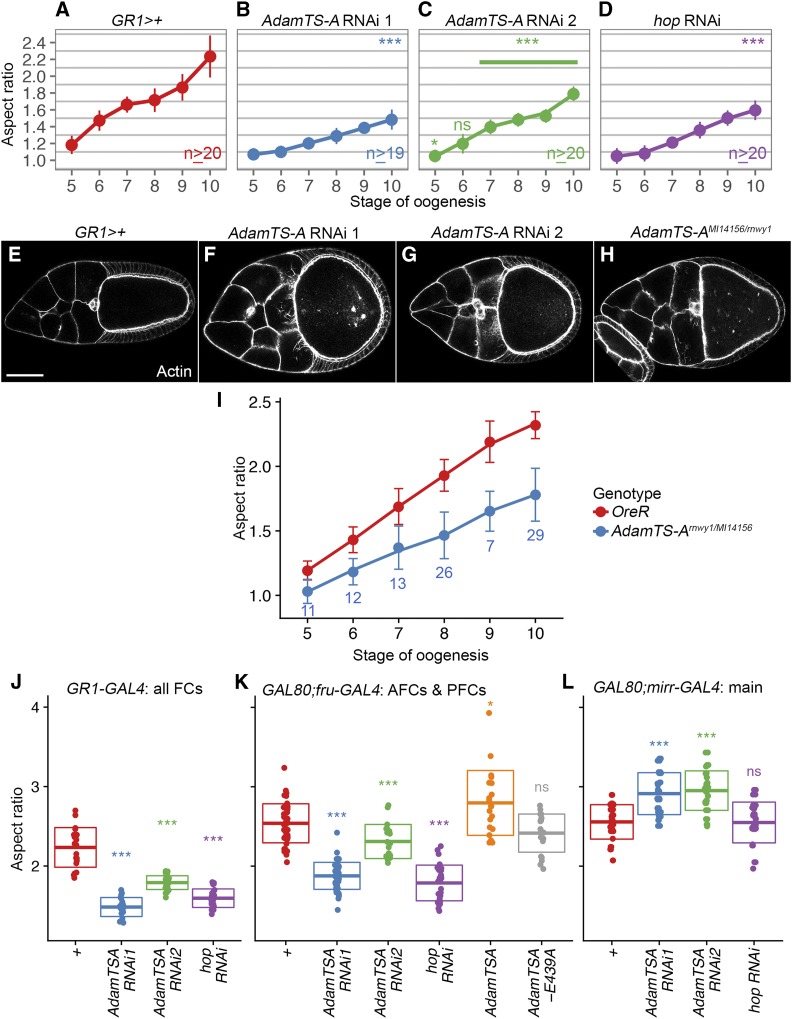
Disrupting *AdamTS-A* produces round egg chambers. A-D) *AdamTS-A* and *hop* RNAi knockdown delay egg chamber elongation beginning at stage five. n values reflect sample sizes for each data point. E-H) Disrupting *AdamTS-A* results in a pronounced round egg chamber phenotype at stage 10. Confocal micrographs of stage 10 egg chambers of following genotypes E) *GR1>+* (*GAL4* only control for comparison to F&G), F) *AdamTS-A* RNAi 1, G) *AdamTS-A* RNAi 2, and H) *AdamTS-A^MI14156/rnwy1^*. Scale bar represents 50 μm. I) *AdamTS-A^MI14156^/rnwy^1^* egg chambers are significantly rounder than *OreR* controls beginning at stage 5. For all *OreR* data points, n ≥ 19. For *AdamTS-A^MI14156/rnwy1^*, n values are indicated in blue. J-L) Quantification of egg chamber aspect ratios at stage 10 demonstrates that J) RNAi knockdown of *AdamTS-A* or *JAK/hop* ubiquitously in the follicle cells using the *GR1-GAL4* driver, or disrupting *AdamTS-A* or *JAK/hop* in the terminal follicle cells using (K) *fru-GAL4* and *GAL80* is sufficient to produce a significantly rounder egg at stage 10. Overexpression of *AdamTS-A* in the terminal follicle cells using a *fru-GAL4* and *GAL80* (J) or RNAi knockdown of *AdamTS-A* in the main body follicle cells using a *mirr-GAL4* and *GAL80* (L) cause hyperelongation. However, expression of a protease-dead form of AdamTS-A (*AdamTS-A^E439A^*) in the terminal follicle cells (K) does not produce hyperelongation. *hop* RNAi knockdown in the poles produces a significant elongation defect (K); however, knockdown in the main body follicle cells does not affect the aspect ratio. All error bars represent standard deviation of means. For all panels, * = *P* < 0.05; *** = *P* < 0.0005, ns = not significant.

To quantify the elongation defect observed in *AdamTS-A* RNAi knockdown egg chambers, the aspect ratio (length:width ratio) was measured at stages 5 to 10 (Figure 6A-C). When *AdamTS-A* RNAi 1 was expressed under the control of the *GR1-GAL4* driver (Gupta and Schüpbach 2003), which produces expression in the FCs starting at stage 4 of oogenesis (Figure S1), elongation defects were already evident at stage 5 of oogenesis (Figure 6B&F). *AdamTS-A* RNAi 2 produced a milder phenotype, but also significantly reduced egg chamber aspect ratios at most stages of oogenesis (Figure 6C&G). Disrupting JAK/STAT signaling is also sufficient to reduce egg chamber elongation beginning at stage 5 (*hop* RNAi; Figure 6D), which is consistent with previously published findings (Alégot *et al.* 2018).

To confirm the phenotypes observed by RNAi knockdown, we tested several different combinations of *AdamTS-A* alleles to find a transheterozygous mutant condition where adults survive and females produce viable ovaries. *AdamTS-A^MI14156^* homozygotes survive, but females usually have no detectable ovaries or very small malformed ovaries with severe packaging defects; consequently, these flies never lay eggs. The *AdamTS-A^rnwy1^* allele is reportedly viable and sub-fertile (Skeath *et al.* 2017), but we were not able to obtain homozygous adult flies, which suggests the allele may have developed a secondary lethal mutation. However, heteroallelic *AdamTS-A^MI14156^*/*AdamTS-A^rnwy1^* females produce ovaries of a more normal size and morphology, and while they have reduced fertility, they occasionally lay eggs. Stage 10 *AdamTS-A^MI14156^*/*AdamTS-A^rnwy1^* egg chambers, like AdamTS-A RNAi egg chambers, have an abnormal shape (Figure 6H). We quantified egg chamber elongation in *AdamTS-A^MI14156^/AdamTS-A^rnwy1^* egg chambers and found that, consistent with our RNAi knockdown studies, heteroallelic egg chambers are also significantly rounder than controls at stages 5 to 10 (Figure 6I, *P* < 0.0005).

Since FISH showed that *AdamTS-A* is enriched at the egg chamber poles as compared to the middle, we wanted to test whether altering *AdamTS-A* expression in the poles – or in the main body follicle cells - changes egg chamber shape. *fru-GAL4* drives expression specifically in the egg chamber terminal follicle cells (the poles; see Figure 1A; Barth *et al.* 2012; Borensztejn *et al.* 2013; Crest *et al.* 2017). *mirr-GAL4* has a complementary expression pattern and is expressed in the main body follicle cells beginning at stage 7 (Isabella and Horne-Badovinac 2015; Crest *et al.* 2017). To validate these drivers, we used them to disrupt JAK/STAT signaling by expressing a *hop/JAK* RNAi line. Consistent with prior studies, disrupting JAK/STAT by knocking down *hop/JAK* ubiquitously (*GR1-GAL4*) or in the terminal follicle cells (*fru-GAL4*) produced rounder stage 10 egg chambers (Figure 6J&K), but knockdown in the main body follicle cells (*mirr-GAL4*), where JAK/STAT is not normally active, did not alter egg chamber shape (Figure 6L).

Interestingly, *AdamTS-A* RNAi knockdown in the terminal follicle cells produced hypoelongation (Figure 6K), but knockdown in the main body follicle cells resulted in hyperelongation (Figure 6L). We also found that ectopic expression of *AdamTS-A* (*UAS-AdamTS-A*; Ismat *et al.* 2013) in the poles produced a modest but significant hyperelongation (Figure 6K). Taken together, these results suggest that proper egg chamber shape requires differential *AdamTS-A* expression along the A/P axis, such that it is enriched in the poles and reduced in the middle. To test whether AdamTS-A’s protease activity is required for its function in egg chamber elongation, the *fru-GAL4* driver was used to express the protease dead form of the protein in the poles (*UAS-AdamTS-A^E439A^*; Ismat *et al.* 2013). Unlike the wildtype form of the protein, the protease dead form did not produce hyperelongation (Figure 6K). This suggests that the protease activity of AdamTS-A promotes egg elongation.

## Discussion

Signaling pathways play a critical role during animal development. They organize the body plan, and also sculpt the tissues and organs of the developing animal. Establishing the embryonic body axes is one of the first and most important events in an animal’s development. In *Drosophila melanogaster* this process begins during oogenesis. In developing egg chambers, special follicle cells called the PFCs signal to establish the A/P axis. These cells are specified by a combination of JAK/STAT and EGFR signaling, but it is unclear which targets of JAK/STAT and EGFR signaling mediate their differentiation or signaling behavior. The experiments described herein aimed to identify target genes activated by these two pathways during development, and to screen potential targets for a function in A/P axis establishment or in other aspects of oogenesis.

We used a gene expression approach to identify established and novel transcripts regulated by JAK/STAT and/or EGFR signaling in the follicle cells. Most known PFC-expressed transcripts were present in the microarray dataset, which demonstrates that our gene expression approach was able to successfully identify PFC-expressed RNAs. Surprisingly, transcripts encoding ECM proteins were overrepresented in our microarray screen dataset, which indicates that JAK/STAT and/or EGFR may regulate the ECM at a transcriptional level. We were also surprised to find genes (*mbs*, *α-spec* and *β-spec*) in the microarray dataset that encode proteins involved in posterior signaling but which are not known to be differentially expressed in the PFCs or regulated by the JAK/STAT or EGFR pathways. Using RNAi, we identified new functions and expression patterns for two genes during *Drosophila* oogenesis. *Sema-1b* is a novel PFC-expressed transcript, and *AdamTS-A* becomes enriched in the AFCs and PFCs and plays a role in egg chamber elongation.

It is important to consider the limitations of the approaches used in this study. From the microarray data we have collected, it is not possible to predict whether particular candidate genes are targets of JAK/STAT and/or EGFR signaling. A second important consideration is that the RNAi knockdown reagents used in our screen may not be ideal tools for disrupting candidate oocyte polarization genes in the FCs. It is well-established that many RNAi lines do not effectively reduce target gene expression, which can produce false negatives (Dietzl *et al.* 2007; Perkins *et al.* 2015). Consistent with this observation, several control RNAi lines directed against genes known to be required for posterior signaling (*i.e.*, ERK, MEK, EGFR, and PP1β, see File S5) were not classified as ‘hits’ using the criteria we established. False positives are also a recurrent problem in RNAi screens. Long RNAi reagents can produce off-target gene knockdown in a sequence-dependent manner by cross-hybridizing to transcripts resembling the desired target. It has also been proposed that some RNAi reagents can produce off-target effects in a sequence-independent manner. It is therefore also important to consider that a few false-positives may be present among the RNAi screen ‘hits’.

### Sema1b is expressed in the PFCs and is regulated by JAK/STAT and EGFR

Experiments using transcriptional and translational reporters both suggest that the signaling protein Sema1b is enriched in the PFCs. Using a translational reporter, we observed that disrupting JAK/STAT or EGFR signaling was sufficient to eliminate Sema1b enrichment in the PFCs. Additionally, when EGFR signaling was ectopically activated, Sema1b became strongly enriched in the anterior FCs (AFCs), where JAK/STAT is active, but not in the main body follicle cells, where JAK/STAT is inactive. This suggests that these pathways act together to promote Sema1b expression in the FCS. We did not test whether JAK/STAT and EGFR function to directly regulate *Sema1b* transcription. It is also not clear whether *Sema1b* has a functional role in the FCs.

*Sema1b* RNAi knockdown produced a strong oocyte polarization phenotype; however, results obtained with our *Sema1b^KO^* CRISPR-generated allele suggest that *Sema1b* is not essential for oocyte polarity. It is possible that the *Sema1b* RNAi knockdown phenotype is the result of off-target gene knockdown, however, there are no predicted off-target genes that would easily explain the *Sema1b* knockdown phenotype. The *Sema1b* RNAi reagents do have a 17 bp region of homology that matches Sema1a; however, disrupting both Sema1a and Sema1b using mutants did not produce a posterior signaling phenotype. There are a number of other Sema domain-containing proteins in the *Drosophila* genome, and it was not feasible to disrupt additional ones in this study. It is also possible that the RNAi pathway or certain RNAi reagents used in our study might function systemically to disrupt oocyte repolarization; however, we did not see a significant polarity problem in our controls or in most RNAi lines that we tested. It is possible that the genetic background used for RNAi sensitizes the oocyte for polarity defects, where knockdown of a gene that is peripherally involved in the process could generate a phenotype that it might not show in a different genetic background. These possibilities could be investigated by generating additional *Sema1b* RNAi knockdown reagents and by testing different genetic backgrounds in the presence of the *Sema1b^KO^* mutation.

### JAK/STAT signaling activates AdamTS-A expression in follicle cells

We showed that *AdamTS-A* is enriched at the poles of the egg chamber at stage 6/7. Ectopic JAK/STAT pathway activity in the FCs substantially increases *AdamTS-A* expression in the main body follicle cells, where it is normally present at lower levels. Furthermore, disruption of JAK/STAT signaling using *hop* RNAi knockdown suggests that JAK/STAT signaling is required to establish the normal pattern of *AdamTS-A* expression during oogenesis.

Our analysis does not permit us to distinguish whether *AdamTS-A* is a direct or indirect target of JAK/STAT signaling in the FCs. Confirmed direct targets of JAK/STAT signaling are bound, often at multiple sites, by Stat92E, the sole *Drosophila* STAT protein (Yan *et al.* 1996). The *AdamTS-A* genomic locus contains a number of predicted Stat92E binding sites. Our analysis of a recent ChIP-Seq dataset (Kudron *et al.* 2018; epic.gs.washington.edu/modERN/) revealed that in the 0-12 h *Drosophila* embryo, eGFP-Stat92E binds at three sites in the first intron and promoter of *AdamTS-A* (eGFP-Stat92E ChIP peaks at the *AdamTS-A* locus are marked in Figure S6). It will also be interesting to investigate whether *AdamTS-A* is regulated by JAK/STAT signaling in the other tissues where it is known to be expressed, such as the caudal visceral mesoderm (CVM), salivary gland, hemocytes, and central nervous system glia (Ismat *et al.* 2013; Skeath *et al.* 2017).

### AdamTS-A promotes egg chamber elongation

We found that *AdamTS-A* RNA is expressed strongly in the terminal follicle cells and more weakly in the main body follicle cells. Disrupting *AdamTS-A* throughout the follicular epithelium or in the terminal FCs should reduce the difference in the expression of *AdamTS-A* along the length of the egg chamber. In contrast, knockdown specifically in the main body FCS or ectopic AdamTS-A expression in the terminal FCs should enhance *AdamTS-A*’s enrichment in the egg chamber poles.

In our study, any perturbations that should enhance differential *AdamTS-A* expression along the A/P axis promote hyperelongation and those that reduce the difference produce hypoelongation, leading to a round egg chamber phenotype. By examining *AdamTS-A* RNAi knockdown and hypomorphic mutant egg chambers, we found that *AdamTS-A* is required during the early stages of egg chamber elongation, which is also when JAK/STAT signaling is required. This leads us to propose a model for AdamTS-A function in egg chamber elongation.

The basement membrane (BM), which forms at the basal side of the follicular epithelium, plays an important role in regulating signaling, growth and egg chamber shape. Egg chambers initially grow isotropically, or evenly, in all directions. However, they begin to elongate at stage 5. Live imaging studies revealed that egg chambers rotate perpendicular to the A/P axis throughout much of oogenesis with rotation occurring most rapidly during stages 5 to 9 of oogenesis (Haigo and Bilder 2011). This timing also corresponds to the most rapid phases of egg chamber elongation (Haigo and Bilder 2011; Cetera *et al.* 2014).

The observation that elongation and rotation coincide led to the proposal that egg chamber rotation promotes elongation (Haigo and Bilder 2011). Consistent with this hypothesis, all genetic perturbations reported to disrupt rotation or to cause off-axis rotation also affect elongation. Proteins involved in rotation include the atypical cadherin Fat2, the ECM receptor Lar, Laminins, Misshapen (Msn; a kinase that regulates integrins), and βPS-integrin/Myospheroid (Mys) (Lewellyn *et al.* 2013; Haigo and Bilder 2011; Viktorinová and Dahmann 2013; Barlan *et al.* 2017; Díaz de la Loza *et al.* 2017.) During rotation, the FCs deposit ColIagen IV (Viking; Vkg) to the BM in fibrils oriented perpendicular to the axis of elongation. Collagen is believed to function as a molecular corset during elongation (Haigo and Bilder 2011). If collagen is disrupted, for example by *ColIV* RNAi knockdown or collagenase treatment, egg chamber elongation is also disrupted. However, it is possible to disrupt elongation without disrupting rotation. For example, ectopic SPARC prevents collagen deposition and disrupts elongation but does not interfere with rotation (Isabella and Horne-Badovinac 2015).

Elongation requires several distinct processes that are temporally overlapping (reviewed in Gates 2012; Cetera and Horne Badovinac 2015). The published literature on AdamTS-A suggests a possible mechanism by which it could direct the growth of the egg chamber. In the central nervous system (CNS), AdamTS-A modifies the basement membrane to constrain and direct tissue growth. In this context. AdamTS-A and Perlecan (Pcan/Trol) act together to promote tissue softness, and act in opposition to Collagen IV and βPS-integrin (Mys), which promote tissue stiffness (Skeath *et al.* 2017). Recent work shows that the second phase of egg chamber elongation requires an asymmetric gradient of basement membrane stiffness, with stiffer matrix at the middle and softer matrix at the poles, and this stiffness matrix appears to require JAK/STAT pathway activity (Crest *et al.* 2017). It is therefore attractive to propose that *AdamTS-A* expression is induced by JAK/STAT signaling at the egg chamber poles, and functions to soften the basement membrane at the poles. All our manipulations that led to an increased difference of *AdamTS-A* at the poles *vs.* the middle of the egg chambers resulted in hyperelongation, whereas manipulations that decreased this difference resulted in hypoelongation. Varying AdamTS-A levels affects Collagen IV levels in the central nervous system, and Collagen IV has been proposed as a possible target for AdamTS-A’s protease activity in the CNS (Skeath *et al.* 2017). The results we obtained by modulating *AdamTS-A* expression in the FCs are consistent with it functioning to promote tissue softness, and ColIV is an attractive possible target for AdamTS-A in the egg chamber; however, further studies are warranted to test this hypothesis.

In summary, we used differential expression analysis to generate a list of 317 potential PFC-enriched genes. Genes in this dataset may also be transcriptionally activated by JAK/STAT and/or EGFR signaling. We have investigated some of these genes for a role in oocyte polarization, but others remain to be tested. Our work suggests that Sema1b is expressed in the PFCs, but it remains unclear whether Sema1b plays a functional role in oogenesis. Our work with *AdamTS-A* demonstrates that our screen also has the potential to uncover novel JAK/STAT and/or EGFR target genes with key functional roles in oogenesis.
